# 3-Dodec­yloxy-2-hydr­oxy-*N*,*N*,*N*-trimethyl­propan-1-aminium bromide

**DOI:** 10.1107/S160053680902635X

**Published:** 2009-07-11

**Authors:** Shizhou Fu, Zengbin Wei, Xilian Wei, Tao Wu

**Affiliations:** a College of Chemistry and Chemical Engineering, Liaocheng University, Shandong 252059, People’s Republic of China

## Abstract

In the title compound, C_18_H_40_NO_2_^+^·Br^−^, the ion pairs formed by the hydrogen-bonded bromide anions and organic cations are arranged into thick layers with the alkyl groups directed to the inside and the trimethyl­aminium groups and the bromide anions situated on the layer surface. The long alkyl chain in the cation adopts an all-*trans* conformation. In the crystal structure, molecules are connected by intermolecular O—H⋯Br hydrogen bonds, forming ionic pairs that are further connected into an extended chain structure *via* C—H⋯O hydrogen-bonding interactions. The crystal is chiral but nearly 90% of atoms in the unit cell are related by a pseudo-inversion center. The crystal shows racemic twinning with a 0.33:0.67 domain ratio.

## Related literature

For related structures, see: Koh *et al.* (1993 ▶). For applications of 3-alk­oxy-2-hydroxy­propyl-*N*,*N*,*N*-trimethyl­propan-1-amin­ium bromides, see: Yin *et al.* (2001 ▶); Zhao *et al.* (1997 ▶). 
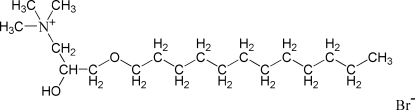


## Experimental

### 

#### Crystal data

C_18_H_40_NO_2_^+^·Br^−^
*M* *_r_* = 382.42
Monoclinic, 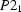

*a* = 6.0476 (11) Å
*b* = 7.5370 (12) Å
*c* = 24.870 (2) Å
β = 94.974 (1)°
*V* = 1129.3 (3) Å^3^
*Z* = 2
Mo *K*α radiation
μ = 1.83 mm^−1^
*T* = 298 K
0.48 × 0.34 × 0.22 mm

#### Data collection

Bruker SMART diffractometer
Absorption correction: multi-scan (*SADABS*; Sheldrick, 1996 ▶) *T*_min_ = 0.474, *T*_max_ = 0.689
5077 measured reflections
3431 independent reflections
1751 reflections with *I* > 2σ(*I*)
*R*_int_ = 0.081

#### Refinement

*R*[*F* ^2^ > 2σ(*F* ^2^)] = 0.063
*wR*(*F* ^2^) = 0.158
*S* = 1.00
3431 reflections
204 parameters
1 restraint
H-atom parameters constrained
Δρ_max_ = 0.54 e Å^−3^
Δρ_min_ = −0.57 e Å^−3^
Absolute structure: Flack (1983 ▶), 1274 Friedel pairs
Flack parameter: 0.33 (3)

### 

Data collection: *SMART* (Siemens, 1996 ▶); cell refinement: *SAINT* (Siemens, 1996 ▶); data reduction: *SAINT*; program(s) used to solve structure: *SHELXS97* (Sheldrick, 2008 ▶); program(s) used to refine structure: *SHELXL97* (Sheldrick, 2008 ▶); molecular graphics: *SHELXTL* (Sheldrick, 2008 ▶); software used to prepare material for publication: *SHELXTL*.

## Supplementary Material

Crystal structure: contains datablocks I, global. DOI: 10.1107/S160053680902635X/gk2214sup1.cif

Structure factors: contains datablocks I. DOI: 10.1107/S160053680902635X/gk2214Isup2.hkl

Additional supplementary materials:  crystallographic information; 3D view; checkCIF report

## Figures and Tables

**Table 1:** Hydrogen-bond geometry (Å, °)

*D*—H⋯*A*	*D*—H	H⋯*A*	*D*⋯*A*	*D*—H⋯*A*
C6—H6*C*⋯O1^i^	0.96	2.27	3.229 (13)	174
O1—H1⋯Br1^ii^	0.82	2.46	3.272 (6)	170

Symmetry codes: (i) 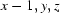; (ii) 
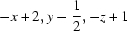
.

**Fig. 1.**
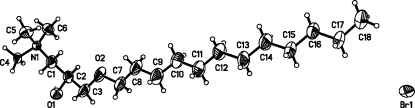
The molecular structure of the title compound, with atom labels and 50% probability displacement ellipsoids.

**Fig. 2.**
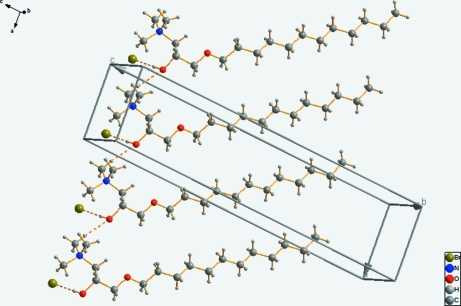
Crystal packing of the title compound, showing one extended chain structure, linked by O—H···Br and C—H···O hydrogen bonds (dashed lines).

## supplementary crystallographic information

### Comment

3-Alkoxy-2-hydroxypropyl-*N*,*N*,*N*-trimethylpropan-1-aminium bromides (RTABs) are a new type of cationic surfactants (Yin *et al.*, 2001).They are used as bacteriocides, emulgents, antistatic agents and pigment-dispersing agents in a variety of industries, such as in the production of cosmetics, pharmaceuticals, and textiles (Zhao *et al.*, 1997). As a contribution to the chemistry of surfactants, we report here the synthesis and crystal structure of the title compound. The asymmetric unit of the title compound (Fig. 1), consists of a 3-dodecyloxy-2-hydroxypropyl-*N*,*N*,*N*- trimethylpropan-1-aminium cations, and a bromide anion. The dodecyl chains of the cations are arranged parallel in one layer and antiparallel in alternate layers, and the C—C—C angles tend to be larger towards the end of the chain. The bond lengths of C3—O2 and C7—O2 are 1.459 (11) and 1.369 (9) Å, respectively. All N—C bond lengths and C—N—C angles are within the usual ranges (Koh *et al.*, 1993). In the crystal, the cations are stacked parallel along the *a* axis (Fig. 2). In the crystal structure, molecules are connected by intermolecular O—H···Br hydrogen bonds forming ionic pairs that are further connected into an extended chain structure via C—H···O hydrogen interactions (Table 1).

### Experimental

The reaction was carried out under nitrogen atmosphere. Trimethylammonium bromide (0.12 mol) and dodecyl glycidyl ether (0.1 mol) were added to a stirred solution of ethanol (100 ml) and stirred at 320 K for 24 h. The resulting clear solution was evaporated under vacuum. Colourless crystals suitable for X-ray analysis were obtained by slow evaporation of a ethyl acetate solution over a period of two weeks. (yield 81%, m.p. 342Kk) Anal. Calcd (%) for C_18_H_40_Br_1_N_1_O_2_ (Mr = 382.42): C, 56.48; H, 10.46; N, 3.66. Found (%): C, 56.43; H, 10.50; N, 3.63.

### Refinement

All H atoms were placed geometrically and treated as riding on their parent atoms with O—H = 0.82 Å, C—H = 0.97 (methylene) Å [*U*_iso_(H) = 1.2*U*_eq_(C)], and C—H = 0.96 (methyl) Å [*U*_iso_(H) = 1.5*U*_eq_(C)]. The crystal was refined as a racemic twin with the 0.33 (3)/0.67 (3) ratio of the twin domains.

### Crystal data

**Table:**

C_18_H_40_NO_2_^+^·Br^−^	*F*(000) = 412
*M**_r_* = 382.42	*D*_x_ = 1.125 Mg m^−^^3^
Monoclinic, *P*2_1_	Mo *K*α radiation, λ = 0.71073 Å
Hall symbol: P 2yb	Cell parameters from 1870 reflections
*a* = 6.0476 (11) Å	θ = 2.5–21.7°
*b* = 7.5370 (12) Å	µ = 1.83 mm^−^^1^
*c* = 24.870 (2) Å	*T* = 298 K
β = 94.974 (1)°	Block, colorless
*V* = 1129.3 (3) Å^3^	0.48 × 0.34 × 0.22 mm
*Z* = 2	

### Data collection

**Table:**

Bruker SMART diffractometer	3431 independent reflections
Radiation source: fine-focus sealed tube	1751 reflections with *I* > 2σ(*I*)
graphite	*R*_int_ = 0.081
Detector resolution: 0 pixels mm^-1^	θ_max_ = 25.0°, θ_min_ = 3.2°
φ and ω scans	*h* = −5→7
Absorption correction: multi-scan (*SADABS*; Sheldrick, 1996)	*k* = −8→8
*T*_min_ = 0.474, *T*_max_ = 0.689	*l* = −27→29
5077 measured reflections	

### Refinement

**Table:**

Refinement on *F*^2^	Secondary atom site location: difference Fourier map
Least-squares matrix: full	Hydrogen site location: inferred from neighbouring sites
*R*[*F*^2^ > 2σ(*F*^2^)] = 0.063	H-atom parameters constrained
*wR*(*F*^2^) = 0.158	*w* = 1/[σ^2^(*F*_o_^2^) + (0.045*P*)^2^] where *P* = (*F*_o_^2^ + 2*F*_c_^2^)/3
*S* = 1.00	(Δ/σ)_max_ = 0.002
3431 reflections	Δρ_max_ = 0.54 e Å^−^^3^
204 parameters	Δρ_min_ = −0.57 e Å^−^^3^
1 restraint	Absolute structure: Flack (1983), 1274 Friedel pairs
Primary atom site location: structure-invariant direct methods	Flack parameter: 0.33 (3)

### Fractional atomic coordinates and isotropic or equivalent isotropic displacement parameters (Å^2^)

**Table:**

	*x*	*y*	*z*	*U*_iso_*/*U*_eq_	
Br1	0.21650 (16)	0.2943 (2)	0.08166 (3)	0.0752 (4)	
N1	1.3970 (9)	0.297 (2)	0.91650 (19)	0.0510 (15)	
O1	1.7912 (10)	0.1699 (9)	0.8526 (2)	0.0742 (18)	
H1	1.7944	0.0836	0.8727	0.089*	
O2	1.3595 (12)	0.2556 (13)	0.7440 (2)	0.095 (4)	
C1	1.4538 (16)	0.337 (3)	0.8576 (3)	0.105 (8)	
H1A	1.3163	0.3636	0.8362	0.126*	
H1B	1.5446	0.4429	0.8585	0.126*	
C2	1.5697 (15)	0.1972 (15)	0.8294 (3)	0.069 (3)	
H2	1.4855	0.0860	0.8283	0.083*	
C3	1.5831 (17)	0.274 (3)	0.7705 (3)	0.118 (5)	
H3A	1.6280	0.3976	0.7719	0.142*	
H3B	1.6888	0.2072	0.7514	0.142*	
C4	1.5977 (11)	0.294 (3)	0.9561 (2)	0.063 (2)	
H4A	1.6733	0.4062	0.9551	0.095*	
H4B	1.6955	0.2010	0.9468	0.095*	
H4C	1.5532	0.2743	0.9917	0.095*	
C5	1.266 (2)	0.467 (2)	0.9240 (5)	0.070 (5)	
H5A	1.3677	0.5631	0.9323	0.105*	
H5B	1.1736	0.4515	0.9530	0.105*	
H5C	1.1758	0.4931	0.8913	0.105*	
C6	1.254 (2)	0.144 (2)	0.9272 (5)	0.081 (5)	
H6A	1.2339	0.1394	0.9650	0.122*	
H6B	1.3237	0.0367	0.9166	0.122*	
H6C	1.1129	0.1574	0.9069	0.122*	
C7	1.3494 (19)	0.260 (3)	0.6888 (3)	0.111 (6)	
H7A	1.4236	0.1554	0.6767	0.133*	
H7B	1.4335	0.3624	0.6784	0.133*	
C8	1.1261 (17)	0.269 (3)	0.6594 (3)	0.100 (4)	
H8A	1.0373	0.3478	0.6796	0.120*	
H8B	1.0610	0.1518	0.6611	0.120*	
C9	1.095 (2)	0.329 (3)	0.5998 (4)	0.126 (6)	
H9A	1.1088	0.4571	0.5986	0.151*	
H9B	1.2151	0.2794	0.5810	0.151*	
C10	0.8776 (18)	0.277 (3)	0.5696 (3)	0.111 (4)	
H10A	0.7585	0.3264	0.5886	0.133*	
H10B	0.8645	0.1488	0.5711	0.133*	
C11	0.841 (2)	0.333 (4)	0.5107 (3)	0.130 (7)	
H11A	0.8769	0.4584	0.5093	0.156*	
H11B	0.9503	0.2710	0.4915	0.156*	
C12	0.626 (2)	0.309 (4)	0.4796 (3)	0.132 (5)	
H12A	0.5771	0.1909	0.4880	0.158*	
H12B	0.5251	0.3907	0.4951	0.158*	
C13	0.585 (2)	0.328 (4)	0.4222 (3)	0.139 (8)	
H13A	0.6986	0.2589	0.4064	0.167*	
H13B	0.6144	0.4513	0.4141	0.167*	
C14	0.3699 (19)	0.282 (3)	0.3913 (3)	0.121 (4)	
H14A	0.3502	0.1547	0.3947	0.145*	
H14B	0.2533	0.3379	0.4097	0.145*	
C15	0.3272 (18)	0.326 (4)	0.3326 (3)	0.129 (7)	
H15A	0.3428	0.4531	0.3290	0.155*	
H15B	0.4445	0.2714	0.3140	0.155*	
C16	0.1111 (19)	0.274 (3)	0.3030 (3)	0.120 (5)	
H16A	0.0889	0.1491	0.3103	0.143*	
H16B	−0.0043	0.3380	0.3197	0.143*	
C17	0.069 (2)	0.298 (4)	0.2446 (4)	0.175 (8)	
H17A	0.1065	0.4204	0.2370	0.210*	
H17B	0.1744	0.2243	0.2279	0.210*	
C18	−0.147 (2)	0.265 (4)	0.2162 (4)	0.171 (10)	
H18A	−0.1791	0.1400	0.2171	0.257*	
H18B	−0.1465	0.3027	0.1794	0.257*	
H18C	−0.2589	0.3290	0.2333	0.257*	

### Atomic displacement parameters (Å^2^)

**Table:**

	*U*^11^	*U*^22^	*U*^33^	*U*^12^	*U*^13^	*U*^23^
Br1	0.0807 (6)	0.0495 (5)	0.0990 (6)	−0.0305 (7)	0.0278 (4)	−0.0123 (8)
N1	0.037 (3)	0.066 (4)	0.050 (3)	0.013 (7)	0.005 (3)	0.003 (8)
O1	0.068 (4)	0.069 (4)	0.086 (4)	0.001 (4)	0.005 (3)	0.006 (3)
O2	0.138 (6)	0.115 (10)	0.030 (3)	0.065 (7)	−0.003 (3)	0.010 (4)
C1	0.065 (6)	0.20 (2)	0.047 (5)	0.023 (11)	−0.010 (5)	−0.007 (9)
C2	0.052 (6)	0.088 (7)	0.067 (7)	0.006 (5)	−0.002 (5)	−0.008 (6)
C3	0.107 (8)	0.210 (16)	0.039 (5)	0.002 (16)	0.006 (5)	0.039 (12)
C4	0.047 (4)	0.085 (6)	0.054 (4)	0.023 (9)	−0.010 (4)	−0.032 (9)
C5	0.072 (9)	0.056 (9)	0.083 (10)	0.046 (7)	0.007 (8)	−0.001 (6)
C6	0.053 (9)	0.070 (10)	0.119 (13)	0.016 (7)	0.001 (8)	−0.037 (9)
C7	0.115 (8)	0.152 (18)	0.066 (6)	0.051 (12)	0.012 (6)	−0.014 (9)
C8	0.124 (9)	0.115 (12)	0.059 (6)	0.018 (11)	0.000 (5)	0.013 (9)
C9	0.157 (11)	0.166 (19)	0.053 (5)	0.010 (14)	0.003 (6)	0.010 (10)
C10	0.133 (9)	0.134 (12)	0.063 (6)	0.020 (14)	−0.006 (6)	−0.016 (11)
C11	0.145 (10)	0.20 (2)	0.046 (6)	−0.005 (14)	0.002 (6)	0.008 (10)
C12	0.162 (10)	0.178 (14)	0.048 (6)	0.035 (18)	−0.025 (6)	−0.010 (14)
C13	0.135 (10)	0.22 (2)	0.053 (6)	−0.046 (16)	−0.019 (6)	0.015 (12)
C14	0.147 (10)	0.152 (12)	0.062 (6)	−0.021 (17)	−0.002 (6)	0.022 (13)
C15	0.120 (9)	0.205 (19)	0.060 (6)	−0.046 (15)	−0.001 (6)	−0.006 (11)
C16	0.141 (10)	0.148 (14)	0.069 (6)	−0.042 (15)	−0.002 (6)	0.004 (12)
C17	0.140 (11)	0.31 (2)	0.069 (7)	0.02 (2)	−0.001 (7)	−0.118 (16)
C18	0.179 (13)	0.26 (3)	0.073 (7)	−0.12 (2)	−0.012 (8)	0.005 (15)

### Geometric parameters (Å, °)

**Table:**

N1—C6	1.48 (2)	C9—C10	1.510 (16)
N1—C4	1.496 (7)	C9—H9A	0.9700
N1—C5	1.524 (18)	C9—H9B	0.9700
N1—C1	1.561 (10)	C10—C11	1.521 (14)
O1—C2	1.426 (10)	C10—H10A	0.9700
O1—H1	0.8200	C10—H10B	0.9700
O2—C7	1.369 (9)	C11—C12	1.463 (16)
O2—C3	1.459 (11)	C11—H11A	0.9700
C1—C2	1.475 (18)	C11—H11B	0.9700
C1—H1A	0.9700	C12—C13	1.435 (11)
C1—H1B	0.9700	C12—H12A	0.9700
C2—C3	1.584 (14)	C12—H12B	0.9700
C2—H2	0.9800	C13—C14	1.495 (15)
C3—H3A	0.9700	C13—H13A	0.9700
C3—H3B	0.9700	C13—H13B	0.9700
C4—H4A	0.9600	C14—C15	1.496 (13)
C4—H4B	0.9600	C14—H14A	0.9700
C4—H4C	0.9600	C14—H14B	0.9700
C5—H5A	0.9600	C15—C16	1.495 (15)
C5—H5B	0.9600	C15—H15A	0.9700
C5—H5C	0.9600	C15—H15B	0.9700
C6—H6A	0.9600	C16—C17	1.465 (13)
C6—H6B	0.9600	C16—H16A	0.9700
C6—H6C	0.9600	C16—H16B	0.9700
C7—C8	1.481 (13)	C17—C18	1.452 (15)
C7—H7A	0.9700	C17—H17A	0.9700
C7—H7B	0.9700	C17—H17B	0.9700
C8—C9	1.544 (13)	C18—H18A	0.9600
C8—H8A	0.9700	C18—H18B	0.9600
C8—H8B	0.9700	C18—H18C	0.9600
			
C6—N1—C4	109.0 (12)	C8—C9—H9A	108.4
C6—N1—C5	108.4 (6)	C10—C9—H9B	108.4
C4—N1—C5	109.3 (11)	C8—C9—H9B	108.4
C6—N1—C1	119.8 (11)	H9A—C9—H9B	107.5
C4—N1—C1	112.8 (6)	C9—C10—C11	117.0 (13)
C5—N1—C1	96.4 (11)	C9—C10—H10A	108.0
C2—O1—H1	109.5	C11—C10—H10A	108.0
C7—O2—C3	114.2 (8)	C9—C10—H10B	108.0
C2—C1—N1	117.5 (15)	C11—C10—H10B	108.0
C2—C1—H1A	107.9	H10A—C10—H10B	107.3
N1—C1—H1A	107.9	C12—C11—C10	121.6 (13)
C2—C1—H1B	107.9	C12—C11—H11A	106.9
N1—C1—H1B	107.9	C10—C11—H11A	106.9
H1A—C1—H1B	107.2	C12—C11—H11B	106.9
O1—C2—C1	112.3 (8)	C10—C11—H11B	106.9
O1—C2—C3	107.7 (8)	H11A—C11—H11B	106.7
C1—C2—C3	104.2 (12)	C13—C12—C11	125.7 (12)
O1—C2—H2	110.8	C13—C12—H12A	105.9
C1—C2—H2	110.8	C11—C12—H12A	105.9
C3—C2—H2	110.8	C13—C12—H12B	105.9
O2—C3—C2	105.3 (9)	C11—C12—H12B	105.9
O2—C3—H3A	110.7	H12A—C12—H12B	106.2
C2—C3—H3A	110.7	C12—C13—C14	123.8 (12)
O2—C3—H3B	110.7	C12—C13—H13A	106.4
C2—C3—H3B	110.7	C14—C13—H13A	106.4
H3A—C3—H3B	108.8	C12—C13—H13B	106.4
N1—C4—H4A	109.5	C14—C13—H13B	106.4
N1—C4—H4B	109.5	H13A—C13—H13B	106.4
H4A—C4—H4B	109.5	C15—C14—C13	121.3 (12)
N1—C4—H4C	109.5	C15—C14—H14A	107.0
H4A—C4—H4C	109.5	C13—C14—H14A	107.0
H4B—C4—H4C	109.5	C15—C14—H14B	107.0
N1—C5—H5A	109.5	C13—C14—H14B	107.0
N1—C5—H5B	109.5	H14A—C14—H14B	106.8
H5A—C5—H5B	109.5	C16—C15—C14	119.5 (13)
N1—C5—H5C	109.5	C16—C15—H15A	107.5
H5A—C5—H5C	109.5	C14—C15—H15A	107.5
H5B—C5—H5C	109.5	C16—C15—H15B	107.5
N1—C6—H6A	109.5	C14—C15—H15B	107.5
N1—C6—H6B	109.5	H15A—C15—H15B	107.0
H6A—C6—H6B	109.5	C17—C16—C15	121.7 (13)
N1—C6—H6C	109.5	C17—C16—H16A	106.9
H6A—C6—H6C	109.5	C15—C16—H16A	106.9
H6B—C6—H6C	109.5	C17—C16—H16B	106.9
O2—C7—C8	117.2 (9)	C15—C16—H16B	106.9
O2—C7—H7A	108.0	H16A—C16—H16B	106.7
C8—C7—H7A	108.0	C18—C17—C16	122.2 (14)
O2—C7—H7B	108.0	C18—C17—H17A	106.8
C8—C7—H7B	108.0	C16—C17—H17A	106.8
H7A—C7—H7B	107.3	C18—C17—H17B	106.8
C7—C8—C9	121.1 (11)	C16—C17—H17B	106.8
C7—C8—H8A	107.1	H17A—C17—H17B	106.6
C9—C8—H8A	107.0	C17—C18—H18A	109.5
C7—C8—H8B	107.1	C17—C18—H18B	109.5
C9—C8—H8B	107.1	H18A—C18—H18B	109.5
H8A—C8—H8B	106.8	C17—C18—H18C	109.5
C10—C9—C8	115.4 (13)	H18A—C18—H18C	109.5
C10—C9—H9A	108.4	H18B—C18—H18C	109.5
			
C6—N1—C1—C2	−59.5 (13)	C7—C8—C9—C10	161.0 (19)
C4—N1—C1—C2	70.9 (17)	C8—C9—C10—C11	−179.9 (18)
C5—N1—C1—C2	−175.0 (11)	C9—C10—C11—C12	−172 (2)
N1—C1—C2—O1	−68.1 (13)	C10—C11—C12—C13	−168 (2)
N1—C1—C2—C3	175.6 (10)	C11—C12—C13—C14	172 (3)
C7—O2—C3—C2	−160.1 (15)	C12—C13—C14—C15	172 (2)
O1—C2—C3—O2	165.6 (11)	C13—C14—C15—C16	179 (2)
C1—C2—C3—O2	−75.0 (16)	C14—C15—C16—C17	−174 (2)
C3—O2—C7—C8	−171.2 (18)	C15—C16—C17—C18	−174 (3)
O2—C7—C8—C9	162.0 (18)		

### Hydrogen-bond geometry (Å, °)

**Table:**

*D*—H···*A*	*D*—H	H···*A*	*D*···*A*	*D*—H···*A*
C6—H6C···O1^i^	0.96	2.27	3.229 (13)	174
O1—H1···Br1^ii^	0.82	2.46	3.272 (6)	170

Symmetry codes: (i) *x*−1, *y*, *z*; (ii) −*x*+2, *y*−1/2, −*z*+1.
